# Supplementary material for: Altered microRNA Transcriptome in Cultured Human Liver Cells upon Infection with Ebola Virus
Source: Int J Mol Sci. 2021 Apr 6;22(7):3792. doi: 10.3390/ijms22073792 (PMC8038836; doi:10.3390/ijms22073792)
Supplement: Supplementary file 1 [file ijms-22-03792-s001.zip › Supplementary_File/C_ GO_Analysis_Results/16-30nt_go_Reston-96h-Huh7_vs_Control-96h-Huh7_down.mature_mirna_targets/MF_Pvalue_tree.pdf]

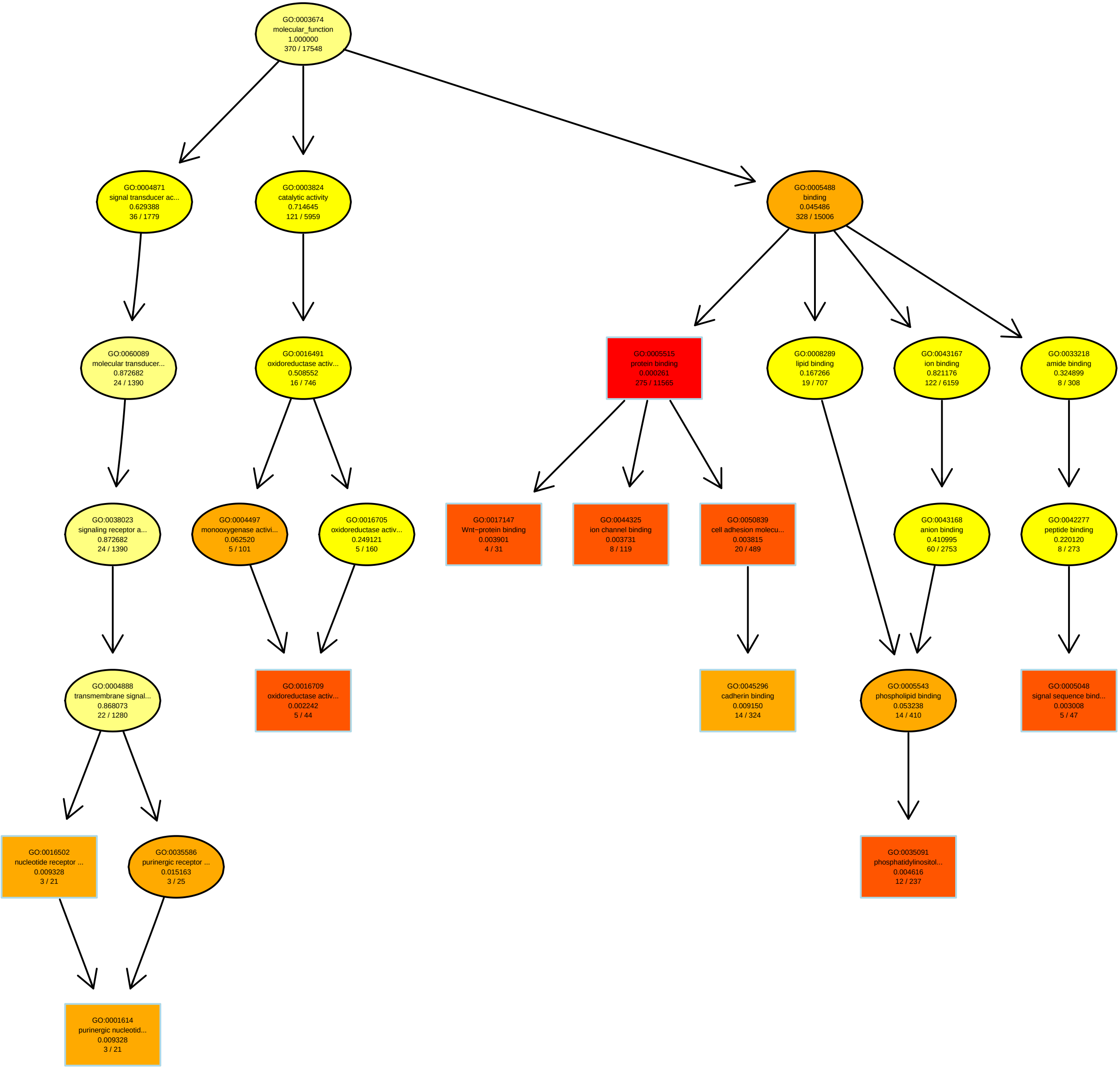
GO:0003674
molecular_function
1.000000
370 / 17548
GO:0004871
signal transducer ac...
0.629388
36 / 1779
GO:0003824
catalytic activity
0.714645
121 / 5959
GO:0005488
binding
0.045486
328 / 15006
GO:0060089
molecular transducer...
0.872682
24 / 1390
GO:0016491
oxidoreductase activ...
0.508552
16 / 746
GO:0005515
protein binding
0.000261
275 / 11565
GO:0008289
lipid binding
0.167266
19 / 707
GO:0043167
ion binding
0.821176
122 / 6159
GO:0033218
amide binding
0.324899
8 / 308
GO:0038023
signaling receptor a...
0.872682
24 / 1390
GO:0004497
monooxygenase activi...
0.062520
5 / 101
GO:0016705
oxidoreductase activ...
0.249121
5 / 160
GO:0017147
Wnt−protein binding
0.003901
4 / 31
GO:0044325
ion channel binding
0.003731
8 / 119
GO:0050839
cell adhesion molecu...
0.003815
20 / 489
GO:0043168
anion binding
0.410995
60 / 2753
GO:0042277
peptide binding
0.220120
8 / 273
GO:0004888
transmembrane signal...
0.868073
22 / 1280
GO:0016709
oxidoreductase activ...
0.002242
5 / 44
GO:0045296
cadherin binding
0.009150
14 / 324
GO:0005543
phospholipid binding
0.053238
14 / 410
GO:0005048
signal sequence bind...
0.003008
5 / 47
GO:0016502
nucleotide receptor ...
0.009328
3 / 21
GO:0035586
purinergic receptor ...
0.015163
3 / 25
GO:0035091
phosphatidylinositol...
0.004616
12 / 237
GO:0001614
purinergic nucleotid...
0.009328
3 / 21
